# Cancer Salivary Biomarkers for Tumours Distant to the Oral Cavity

**DOI:** 10.3390/ijms17091531

**Published:** 2016-09-12

**Authors:** Óscar Rapado-González, Blanca Majem, Laura Muinelo-Romay, Rafa López-López, María Mercedes Suarez-Cunqueiro

**Affiliations:** 1 Department of Surgery and Surgical Medical Specialties, Medicine and Dentistry School, University of Santiago de Compostela, Santiago de Compostela 15782, Spain; oscarr16691@gmail.com; 2 Biomedical Research Unit in Gynecology, Vall Hebron Research Institute (VHIR) and University Hospital, Autonomous University of Barcelona, Barcelona 08035, Spain; blanca.majem@vhir.org; 3 Liquid Biopsy Analysis Unit, Health Research Institute of Santiago (IDIS), Complexo Hospitalario Universitario de Santiago de Compostela (SERGAS), Santiago de Compostela 15706, Spain; lmuirom@gmail.com (L.M.-R.); rafa.lopez.lopez@gmail.com (R.L.-L.); 4 Translational Medical Oncology, Health Research Institute of Santiago (IDIS), Complexo Hospitalario Universitario de Santiago de Compostela (SERGAS), Santiago de Compostela 15706, Spain

**Keywords:** non-invasive diagnosis, saliva, systemic diseases, biomarkers, cancer

## Abstract

The analysis of saliva as a diagnostic approach for systemic diseases was proposed just two decades ago, but recently great interest in the field has emerged because of its revolutionary potential as a liquid biopsy and its usefulness as a non-invasive sampling method. Multiple molecules isolated in saliva have been proposed as cancer biomarkers for diagnosis, prognosis, drug monitoring and pharmacogenetic studies. In this review, we focus on the current status of the salivary diagnostic biomarkers for different cancers distant to the oral cavity, noting their potential use in the clinic and their applicability in personalising cancer therapies.

## 1. Introduction

Currently, a large proportion of cancer cases are still diagnosed at the advanced stage due to a wide variety of personal, socioeconomic and medical reasons [1]. While the rates of survival for certain cancers have improved, overall they are still quite low. Hence, there is an urgent need for further improvement of cancer survival, being one of the main goals of the World Cancer Declaration issued by the Union for International Cancer Control. Like the proverbial elephant in the room, cancer is far too large a problem to be safely ignored [2]. Therefore, any new diagnostic tool that can potentially improve cancer diagnosis and disease monitoring implies an improvement in overall survival and is worth considering.

The analysis of saliva as a diagnostic approach for systemic diseases was proposed just two decades ago, but great interest in the field has emerged recently because of its revolutionary potential as a liquid biopsy. Saliva is called the mirror of the body [3], as it is considered an ultra-filtrate of the blood [4] and because its composition changes under different pathological conditions. Currently, five diagnostic alphabets have been characterised in saliva: proteome, transcriptome, microRNA (miRNA), metabolome and microbiome [5]. More recently, piwi-interacting RNAs (piRNAs), circular RNAs (circRNAs) and other non-coding RNAs (ncRNAs) have also been described as a new landscape of salivary RNAs [6]. Multiple molecules isolated from saliva can be used as cancer biomarkers for diagnosis, prognosis, drug monitoring and pharmacogenetic studies [7].

The attractiveness of saliva for diagnosis is based on the fact that it can be obtained easily, repeatedly and non-invasively. Moreover, storage is simple and no special equipment is needed for sample collection, making it very cost-effective [8].

Here, we review the current status of the salivary diagnostic biomarkers for different cancers distant to the oral cavity, noting their potential use in the clinic and their applicability.

## 2. Systemic Cancer and Saliva

There is growing evidence of the value of saliva for detecting not only benign pathological conditions but also malignancies distant to the oral cavity. Indeed, several molecules have been isolated from saliva, demonstrating sufficient sensitivity and specificity [9,10] as diagnostic biomarkers for different remote cancers [11,12,13,14].

In this sense, many efforts have been made in accordance to establish the methodology for the collection, processing and storage of saliva [8], as well as different approaches for stabilising and preventing RNA and protein degradation [15,16,17]. Importantly, several groups from the extracellular RNA (exRNA) consortium of the National Institutes of Health (NIH) have combined efforts to develop methods and protocols for manipulating body fluids, including saliva [18], where they described technical aspects from sample collection to analysis, pointing out technical aspects regarding additional steps for improving the quality of the analysed samples (i.e., DNase treatment, RNA precipitation, etc.) [18]. Despite these efforts, saliva remains a complex body fluid because more than 50% of the molecules arise from bacterial loading into the saliva [19], but novel technologies and incessant research have greatly improved the characterisation of the salivary landscape [6].

**Figure 1:**
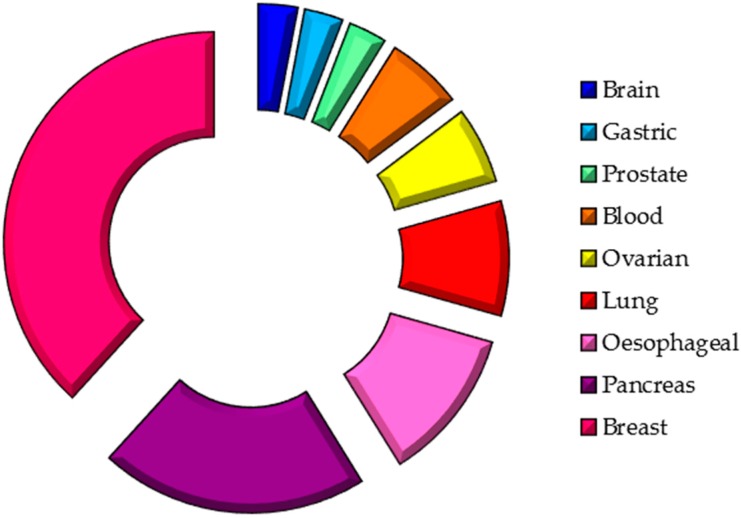
Salivary biomarker—sites of origin.

Thus, saliva is a non-invasive diagnostic body fluid and a potential source of biomarkers. Although many studies have been performed pursuing the same aim (Table 1), there is still a need for further studies to validate its clinical application. To date, many biomarkers have been described for a wide variety of cancers distant to the oral cavity (Figure 1), which are summarised below.

### 2.1. Brain Cancer

According to the latest estimated data published by the GLOBOCAN project, 139,608 and 116,605 new brain cancer cases were diagnosed in males and females, respectively, worldwide in 2012 [48]. Glioblastoma is the most frequent brain cancer, and although primary malignant brain tumours are scarce, survival is poor in patients with glioblastoma [49].

Due to its implication in cell growth and proliferation, oxidative stress is a factor in the development of brain cancer [50]. Although oxidative stress has been studied in many cancers as a pro-tumourigenic factor [51], brain tissue is considered abnormally sensitive to oxidative damage because of its high oxygen consumption rate, high lipid content and relatively low antioxidant defences compared to other tissues. Suma et al. [20] evaluated ferric reducing antioxidant power (FRAP) and protein thiol levels as possible markers of oxidative stress and predictors of disease prognosis in saliva. On one hand, the mean salivary FRAP values were significantly lower among patients with benign and malignant brain tumours. On the other hand, the mean protein thiol values were significantly higher among patients with benign and malignant brain tumours. However, no significant differences in salivary FRAP or protein thiols were observed between the benign and malignant tumour groups, although these two markers appear to be good indicators of oxidative stress status in the saliva of patients with brain tumours. Thus, a possible window of opportunity is presented in the form of a non-invasive diagnostic technique in these inaccessible tumours, accompanied by the possible therapeutic approach of using antioxidants that may slow or even prevent the development of brain cancer.

### 2.2. Pancreatic Cancer

Pancreatic cancer has a five-year relative survival rate of 7% [52], and novel diagnostic tests are urgently needed. Several studies have shown that mRNA and miRNAs can be potential diagnostic and prognostic markers [53] in patients with pancreatic cancer, being characterised in blood [54], stools [55], pancreatic juice [56] and the tumours themselves [57]. Interestingly, the presence of these molecules has also been described in saliva. Zhang et al. [10] found salivary transcriptomic biomarkers associated with resectable pancreatic cancer. The authors used a logistic regression model that included four mRNAs (*KRAS*, *MBD3L2*, *ACRV1*, and *DPM1*) with 90% sensitivity and 95% specificity. Hence, these biomarkers could discriminate between patients with and without pancreatic cancer (i.e., patients with chronic pancreatitis or healthy controls). The high sensitivity and specificity suggest that these biomarkers could be useful for performing a screening test for pancreatic cancer.

A number of studies have shown the important role of miRNAs in the evolutionary course of pancreatic tumours, where they influence several biologic pathways related to tumour progression [58]. Currently, miRNAs are considered emergent biomarkers because they have advantages such as greater stability in body fluids, easier screening and more accurate quantitation [59,60]. A recent study [12] of salivary biomarkers found significant downregulation of miR-3679-5p and upregulation of miR-940 between a pancreatic cancer group and two non-cancer control groups (healthy controls and benign pancreatic tumours). Logistic regression models were used to validate the discriminatory power of the two miRNAs between the cancer group and the two control groups: cancer vs. healthy controls presented 72.5% sensitivity and 70% specificity; cancer vs. benign pancreatic tumours presented 62.5% sensitivity and 80.0% specificity; and cancer vs. non-cancer presented 70% sensitivity and 70% specificity. The higher positive and negative predictive values of these models confirmed the enormous discriminatory potential of miRNAs for detecting pancreatic cancer.

Recently, Gao et al. [23] identified a number of miRNAs as saliva biomarkers for detecting pancreatic cancer. The authors analysed the expression of 384 specific cancer-related miRNAs in the saliva of healthy controls and patients with pancreatic cancer and correlated the results with various ZHENG disease conditions, which is a cornerstone of traditional Chinese medicine. They found five upregulated miRNAs (miR-17, miR-21, miR-181a, miR-181b, and miR-196a), for which there was differential expression between the patients with pancreatic cancer and the healthy individuals. Of these, four (miR-17, miR-21, miR-181b, miR-196a) also had significantly different expression levels in three ZHENG conditions.

Besides the characterisation of pancreatic cancer-related miRNAs in saliva, Sugimoto et al. [21] conducted a metabolomic study in patients with pancreatic cancer. The results showed that eight metabolites were specific for the cancer group, and receiver operating characteristic (ROC) curve analysis indicated high discriminatory power (area under the ROC curve (*AUC*) = 0.944). The authors believed that further studies comparing the metabolomic profile between saliva, blood and tumour tissue would help improve understanding of pancreatic cancer progression and follow-up from a therapeutic perspective, as well as understanding of the biology of each tumour biomarker in saliva. In this context, it is important to mention that metabolic profiles have been analysed in plasma for the early detection of pancreatic cancer. Xie et al. [61] developed and tested a logistic regression model formed by a panel of five metabolites with 77.4% sensitivity and 75.8% specificity (*AUC* = 0.835), indicating the diagnostic potential of plasma metabolic biomarkers.

Similarly, Lau et al. [62] studied the role of pancreatic cancer-derived exosomes in salivary biomarker development, basing their studies on the hypothesis that cancer-related mRNAs are the cargo of vesicles and travel to the circulation and reaching the saliva where they have a potential role as tumour biomarkers. Their study is an in vivo examination of the mechanism of salivary biomarker development in a systemic disease context, reporting that suppressing exosome biogenesis results in the ablation of discriminatory salivary biomarker signatures.

Numerous studies have evaluated circulating miRNAs in the plasma of patients with pancreatic cancer. In these patients, Wang et al. [63] observed upregulated levels of miR-21, miR-210, miR-155 and miR-196a. The combination of these miRNAs discriminates patients with and without cancer with 64% sensitivity and 89% specificity. In another study, Abue et al. [64] reported significantly higher expression levels of miR-483-3p and miR-21 in patients with pancreatic cancer compared to the control group. Importantly, differential expression of miRNAs between tissue and plasma has been reported in patients with pancreatic cancer [65]. Thus, miR-21, miR-205, miR-155, miR-31, miR-203 and miR-214 are significantly upregulated in pancreatic tumour tissue while only miR-129-2-3p is significantly downregulated in tumour tissue. However, miR-205, miR-203 and miR-214 are not overexpressed in plasma from patients with cancer as compared to that from healthy individuals [65]. The most recent transcriptomic study of salivary biomarkers in pancreatic cancer was carried out by Humeau et al. [24], where they found that four miRNAs (miR-21, miR-23a, miR-23b, and miR-29c) were significantly expressed in the saliva of patients with pancreatic cancer as compared to the control group, showing 71.4%, 85.7%, 85.7% and 57% sensitivity, respectively, and 100% specificity. miR-23a and miR-23b were overexpressed in the saliva samples of patients with pancreatic cancer precursor lesions. Moreover, miR-210 and let-7c were overexpressed in patients with pancreatitis with respect to the control group, and miR-216 was upregulated in the patients with cancer as compared to the pancreatitis group. Interestingly, the authors also validated miR-21 in vivo by reproducing pancreatic xenografts in mice and analysing miRNA levels in the saliva of the mice. Therefore, their system reveals that salivary miRNA detection precedes systemic detection of cancer cell markers.

Interestingly, several studies have analysed the role of bacteria as reliable disease-specific salivary biomarkers [66]. Farrell et al. [22] compared the salivary microbiome between a pancreatic cancer group and a non-cancer group in pellet fractions. They found two microbial (*Neisseria elongata* and *Streptococcus mitis*) species that showed significant differences between the patients with pancreatic cancer and the healthy controls. A regression model including these microbial biomarkers yielded 96.4% sensitivity and 82.1% specificity with an *AUC* = 0.90. Moreover, the levels of microbial species (*Granulicatella adiacens* and *S. mitis*) were significantly different between pancreatic cancer vs. chronic pancreatitis and pancreatic cancer vs. non-cancer groups. However, Torres et al. [25] did not find significant differences between healthy controls and patients with cancer in the relative abundance of *S. mitis* or *G. adiacens*. They observed a significantly higher ratio of *Leptotrichia* sp. to *Porphyromonas* sp. in the saliva of patients with pancreatic cancer with respect to the saliva of healthy patients or those with other diseases.

### 2.3. Lung Cancer

Despite significant efforts, lung cancer continues to be the most lethal cancer [48]. Early detection is vital for improving survival and quality of life [67], but lung cancer screening tools are often invasive and lack sensitivity and specificity. A number of researchers have investigated saliva as a specific biomarker of the disease [9,26]. For example, in a salivary transcriptomic analysis involving the pre-validation of seven upregulated genes, Zhang et al. [26] found significant differences between patients with lung cancer and healthy controls. They developed a logistic regression model that included five genes (*CCNI*, *EGFR*, *FGF19*, *FRS2*, and *GREB1*) and obtained an *AUC* value of 0.925 with 93.75% sensitivity and 82.81% specificity, demonstrating the discriminatory power of these biomarkers for detecting lung cancer. Indeed, Zhang et al. performed microarray studies on other cancers [10,13], reporting no alteration of the above mRNAs and therefore confirming the specificity of the detected mRNA signature to lung cancer.

A proteomic study of saliva by Xiao et al. [9] found that the levels of three proteins (HP, AZGP1, and human calprotectin) were significantly higher among patients with lung cancer than among healthy controls. A regression model including the three proteins yielded 88.5% sensitivity and 92.3% specificity with an *AUC* of 0.90. The high positive and negative predictive values reported are indicative of the discriminatory power of these protein biomarkers. In addition, the authors found high concentrations of the three proteins in non-small cell lung cancer cell lines. In another proteomic study, Li et al. [27] identified other salivary biomarkers using surface-enhanced Raman spectroscopy. Nine biomolecular peaks presented significant differences between the control and lung cancer groups. The high sensitivity and specificity (78% and 83%, respectively) of the amino acids and nucleic acid bases indicate the potential of this technique for the salivary diagnosis of lung cancer.

Finally, Wei et al. [68] developed a multiplexible electrochemical sensor that can detect epidermal growth factor receptor (*EGFR*) mutations directly in body fluids, including saliva. *EGFR* mutations predict the sensitivity to *EGFR*-targeted therapy, and currently the detection of these mutations is mainly based on tissue biopsy, which is invasive, expensive and time-consuming. The ROC analysis indicated that the system detected exon 19 deletion and the L858R mutation with *AUC* of 0.94 and 0.96, respectively, suggesting that the presence of DNA in saliva is also a good biomarker for disease monitoring, and in this case, for therapy selection.

### 2.4. Gastric Cancer

Gastric cancer is the third most lethal cancer in the world, ranking below lung and liver cancer [48]. Currently, endoscopy is the only available method for detecting gastric-related diseases, including benign and malignant diseases. Hence, the search for minimally invasive diagnostic methods with easy access that can be performed by any clinician without specific experience is necessary. However, the low sensitivity and specificity of diagnostic techniques requires the identification of powerful biomarkers to detect the disease at the early stages and to avoid unnecessary endoscopies. Despite much effort being made in the search for diagnostic biomarkers in serum [69], there is only one study related to saliva and diagnostic biomarkers for detecting gastric cancer. Wu et al. [28] identified four salivary proteins by mass spectrometry that were significantly different between the gastric cancer and control groups. These four salivary markers had 95.65% sensitivity and 100% specificity. Specifically, the 1472.78-Da peptide showed a high level of expression in the saliva of patients with cancer, and the authors suggested its possible role in tumour pathogenesis.

### 2.5. Oesophageal Cancer

Oesophageal cancer is the eighth most common cancer worldwide and its incidence in men is double that in women, with very poor overall survival [48]. Various studies have identified aberrant expression of miRNAs in the serum [70], plasma [71] and tumoural tissue [72] from patients with oesophageal cancer. In the discovery phase of their study, Xie et al. [11] found that five miRNAs (miR-144, miR-10b, miR-451, miR-486-5p, and miR-634) were altered in the saliva of patients with oesophageal cancer. Apart of these miRNAs, they also selected miR-21 for the validation phase, as many studies have reported its aberrant expression in plasma and tissue from patients with oesophageal cancer. Interestingly, the expression levels of miR-144, miR-10b and miR-451 were significantly correlated between whole saliva and supernatant saliva, revealing no significant difference in the discriminatory power of all validated miRNAs in both saliva fractions. In whole saliva, miR-10b, miR-144 and miR-451 showed 89.7%, 92.3% and 84.6% sensitivity, respectively, and 57.9%, 47.4% and 57.9% specificity, respectively. In saliva supernatant, miR-10b, miR-144, miR-21 and miR-451, showed 79.5%, 43.6%, 89.7% and 51.3% sensitivity, respectively, and 57.9%, 89.5%, 47.4% and 84.2% specificity, respectively.

Xie et al. [29] analysed miR-21 expression levels in saliva supernatant. MiR-21 was significantly upregulated in the cancer group as compared to the control group and was not significantly correlated with cancer stage, differentiation and nodal metastasis. Ye et al. [31] compared the diagnostic value of salivary and plasma miR-21 in 100 patients with oesophageal cancer (Stage I, 50; Stage II, 50) and 50 healthy controls. They also found a significant positive correlation between the expression of plasma and salivary miR-21 with comparable diagnostic value, demonstrating the potential of saliva for detecting oesophageal cancer. In addition, Li et al. [73] evaluated the expression levels of nine miRNAs, including miR-21, in the plasma of patients with oesophageal cancer and their association with clinicopathological features. They found higher levels of miR-21 in patients with oesophageal squamous cell carcinoma than in healthy volunteers, where these levels correlated with poor prognosis. Returning to saliva samples, it is important to mention that Wu et al. [30] investigated the diagnostic value of miR-144 in the saliva samples of patients with oesophageal cancer, finding that it was highly expressed in the samples, indicating its potential as a genetic marker for the early detection of oesophageal cancer.

### 2.6. Breast Cancer

Breast cancer is the most common cancer among women and represents the most frequent cause of cancer death among women in less developed countries [48]. The development of biomarkers for early detection could help overcome the limitations of current diagnostic methods [74].

The first markers analysed were EGFR, p53, cathepsin D, c-*erb*B-2 (HER2/neu) and CA15-3 [32,33,34]. Navarro et al. [32] found significantly elevated salivary EGF concentrations in active and follow-up patients with breast cancer; however, the opposite tendency was found in plasma, and there was no correlation between plasma and salivary EGF values. Streckfus et al. [33,34] found significantly higher c-*erb*B-2 and CA15-3 in the saliva and serum in the cancer group as compared to the non-cancer groups. In addition, Bigler et al. [75] evaluated the usefulness of c-*erb*B-2 for monitoring breast cancer and found significant differences in pre- and post-therapy salivary concentrations of c-*erb*B-2, suggesting its potential for tracking treatment and guiding therapy.

Due to the heterogeneity of c-*erb*B-2 expression, Streckfus et al. [36] performed more exhaustive proteomic research. They analysed the salivary proteomic profiles of invasive breast cancer, ductal carcinoma in situ and fibroadenomas. They identified a total 130 proteins, of which 40 had significant differential expression in the malignant and benign group vs. the control group. A similar study analysing salivary protein profiles in 10 women with Stage IIa and Stage IIb invasive ductal carcinoma identified a total 174 proteins, of which 55 were found at both stages. Interestingly, 20 and 28 proteins were specific for Stage IIa and Stage IIb disease, respectively [38]. More recently, salivary protein profiles were compared among patients with breast cancer with positive or negative c-*erb*B-2 receptor, identifying a total 188 salivary proteins in both groups, of which 34 upregulated proteins and 37 downregulated proteins were significantly differentially expressed. Most salivary protein alterations secondary to c-*erb*B-2 receptor status are thought to be involved in the immune response, metabolism and cell structure [76].

Brooks et al. [35] analysed vascular endothelial growth factor (VEGF), EGF and carcinoembryonic antigen (CEA) in 98 saliva samples from patients with breast cancer and from controls. The saliva of the patients had significantly higher levels of these proteins. The logistic model combining VEGF and EGF achieved 83% sensitivity and 74% specificity. There were salivary and serum detectable concentrations of CA15-3 in the early-stage cancer group as compared to the control group [37], where there were higher salivary and serum values in Stage II compared to Stage I disease. Despite the lower salivary flow rate in the patients with cancer, there was a significant positive correlation between serum and saliva CA15-3 concentrations as well as between serum CA15-3 concentrations and salivary CA15-3 output, again showing the potential of saliva as a diagnostic body fluid. On the contrary, Laidi et al. [77] analysed CA15-3 in the saliva and serum of patients with breast cancer and a control group, and found no significant difference. However, they found a significant positive correlation between salivary and serum CA15-3, suggesting that saliva could be used as an alternative to blood. Regarding these markers in plasma, Zajkowska et al. [78] analysed the diagnostic power of VEGF, macrophage colony-stimulating factor (M-CSF) and CA15-3 for detecting breast cancer in a recent study. They observed significantly high sensitivity and specificity of VEGF, especially in early-stage cancer. Ławicki et al. [79] evaluated the levels of VEGF, matrix metalloproteinase-9 (MMP-9) and tissue inhibitor of metalloproteinase-1 (TIMP-1) in patients diagnosed with breast cancer. They found significant higher concentrations of VEGF in the patients with respect to women without cancer. The combination of VEGF or MMP-9 with CA15-3 showed high sensitivity and specificity in Stage III and IV disease. Moreover, VEGF and the combination of VEGF and CA15-3 showed high diagnostic value in early breast cancer [79].

A study compiling salivary transcriptomic and proteomic approaches [13] analysed a total 113 saliva samples to provide biomarkers for breast cancer detection. Significant differences were observed in the salivary transcriptomic and proteomic profiles between patients with breast cancer and the controls. The combination of eight salivary transcriptomic biomarkers (CSTA, TPT1, IGF2BPI, GRM1, GRIK1, H6PD, MDM4, S100A8) and one salivary proteomic biomarker (CA6) enabled the diagnosis of breast cancer with high accuracy (92%), with 83% sensitivity and 97% specificity, respectively.

Efforts to identify salivary biomarkers in patients with breast cancer have been made not only at proteomic and transcriptomic level but also at metabolomic level. Sugimoto et al. [21] identified 28 metabolites in a sample cohort of 30 saliva samples from patients with breast cancer. Although significant differences were found in several metabolites among the patients and healthy controls, none was specific for breast cancer. Multiple logistic regression models using 14 metabolites yielded a cross-validation AUC of 0.881. In another metabolomic study, Takayama et al. [42] investigated the presence of polyamines in the saliva of patients with breast cancer and healthy controls. Numerous polyamines (SPM, Ac-SPM, N8-Ac-SPD, DAc-SPM, N1-Ac-SPD, DAc-SPD, CAD, and Ac-PUT) were present in significantly higher concentrations in the cancer group with respect to the control group, while two polyamines (ORN and PUT) were present in higher concentrations in the control group. A diagnostic equation for discriminating patients with breast cancer patients from those without cancer was obtained, with a concordance rate of 88% in the validation set. Furthermore, the authors determined the post-surgery concentration of the polyamines in the cancer group and developed a discriminant analysis formed by six polyamines (N8-Ac-SPD, N1-Ac-SPD, CAD, DAc-SPD, PUT, and Ac-PUT) with a concordance rate of 70% between the pre- and post-surgical measurements. Indeed, an exhaustive analysis showed decreased N1-Ac-SPD and increased N8-Ac-SPD. The ratio of N8-Ac-SPD/(N1-Ac-SPD + N8-Ac-SPD) showed nearly 80% sensitivity and specificity, suggesting its potential as an index of healthy status after surgical treatment.

In a study involving patients with breast cancer, Cao et al. [39] analysed the salivary proteome using isobaric tags for relative and absolute quantitation (iTRAQ) coupled with liquid chromatography tandem mass spectrometry (LC-MS/MS). Nine proteins with 1.5-fold upregulation or downregulation were associated with cancer with respect to the healthy controls in the study. Moreover, a pilot study using surface-enhanced laser desorption/ionisation (SELDI) MS performed by Streckfus et al. [80] on six saliva samples identified proteins with higher intensity in patients with in situ breast carcinoma compared with healthy women, suggesting that this technology may be a very useful tool in the development of salivary biomarkers.

In a recent study of salivary biomarkers in patients with breast cancer [40], Cheng et al. analysed salivary free amino acids (SFAAs), showing that there were significant differences between early-stage breast cancer (Stage I and II), late-stage breast cancer (Stage III and IV) and healthy controls. Of all the SFAAs identified, 15 showed significant differences between early-stage breast cancer and the control group. A final model that included proline (Pro), threonine (Thr) and histidine (His) yielded 88.2% sensitivity and 85.7% specificity for detecting breast cancer.

Finally, Woods et al. [41] analysed the concentrations of lung resistant protein (LRP) in healthy controls and patients with Stage I breast cancer. They observed significantly higher average intensities at the 110, 85 and 75 kDa in the cancer group as compared to the control group. The authors also determined the risk ratio for Stage I breast cancer, showing significantly increased risk at the LRP antibody-reactive bands: 110, 85 and 75 kDa. Overall, the results showed that LRP was present in significantly higher concentrations among the subjects with breast cancer as compared to healthy women, suggesting it is a novel prognostic indicator for carcinoma of the breast.

### 2.7. Prostate Cancer

Prostate cancer is the second leading cause of cancer death in men after lung and bronchial cancer [52]. Currently, histopathological analysis (Gleason score) and serum prostate-specific antigen (PSA) levels are determinant keys for therapeutic decision in prostate cancer. However, PSA has some weaknesses as a biomarker, where it is also increased in benign prostatic hyperplasia, its expression levels can be similar in indolent and aggressive prostate cancer and it often fails to indicate patient response to a given treatment accurately. Despite the current controversy, PSA is still used as a tumour marker in prostate cancer [81], but is also associated with circulating plasma miRNAs [82]. Several studies have analysed saliva PSA levels, comparing them with that of serum [43,83,84]. Ayatollahi et al. [83] studied a cohort of healthy men and determined that salivary levels of total and free PSA were significantly lower than serological levels. However, the ratios of free total PSA and serum total PSA were significantly correlated in both body fluids.

Interestingly, Shiiki et al. [43] analysed PSA levels in an animal model and in human samples. In the animal model, they analysed PSA concentrations in serum, saliva and the submandibular gland, and noted that serum PSA concentration was related with tumour size, but no correlation between salivary and serological PSA was found. However, the authors observed a weak correlation between serum PSA and the intra-tissue PSA concentration of the submandibular gland; indeed, the control group do not show PSA concentrations in the serum and saliva, nor in the submandibular gland tissue. The study on patients with prostate cancer demonstrated significant differences in salivary PSA concentration between patients with low and high serum PSA concentrations. A significant linear relationship between salivary PSA and serum PSA was determined in the high serum PSA group.

Unlike previous studies only analysing patients with prostate cancer and/or healthy individuals [43,83], Turan et al. [84] analysed PSA levels in three groups: patients with prostate cancer, patients with benign prostatic hyperplasia and healthy individuals (control). A significant difference between patients with prostatic disease (benign or malignant) and the control group was found only for serum free PSA levels, total PSA levels and the free/total PSA ratios. Furthermore, no significant correlation was observed between salivary and serological PSA levels among the groups, suggesting that further studies are needed to discover good candidate salivary biomarkers for detecting prostate cancer.

### 2.8. Leukemic Cancer

Leukaemia is the most common cancer in children, and the incidence of acute lymphoblastic leukaemia (ALL) is highest [85]. Currently, blood and bone marrow are used for diagnosis and therefore the development of non-invasive tools for diagnosis and disease monitoring are of fundamental importance due to the vulnerable population affected.

A genomic study by Chen et al. [44] analysed 30 common fusion gene transcripts that are the hallmark of leukaemia (i.e., *BCR*-*ABL* among others) in the saliva samples of seven leukemic patients and 20 healthy individuals. The results showed concordance between the fusion genes detected in the saliva samples with those in the bone marrow. In addition, fusion transcripts were detected in whole saliva and the levels remained stable when the saliva was stored at room temperature with their settled device for four weeks, indicating a new, accurate and reproducible non-invasive method for detecting leukemic cancer in children.

Recently, Gershan et al. [86] performed a proteomic study to determine the concentration range of salivary neuropeptides in three pools of salivary samples, including children with ALL. They did not find significant differences in salivary cortisol and salivary neuropeptide (sNP) in children without intervention, but they observed a close correlation between serum and salivary vasoactive intestinal polypeptide (VIP). Moreover, in children with ALL, they attempted to correlate pre- and post-intervention sNP concentrations with the autonomic nervous system (ANS) response. They found decreased salivary calcitonin gene-related peptide (CGRP) and VIP after intervention, with the former reaching statistical significance, suggesting the potential role of sNPs as non-invasive biomarkers for integrated therapies in paediatrics.

In the same line, Guerra et al. [45] monitored saliva from patients with childhood cancer with or without antineoplastic treatment at immunological, biochemical and hormone level. They observed that, regardless of treatment, salivary alkaline phosphatase (ALP) levels were significantly higher in the cancer group than in the control group. However, urea concentrations were significantly lower in treated patients compared to the non-treated patients and the controls. Interestingly, they postulated that antineoplastic treatment increases glucose levels and decreases insulin concentrations, reinforcing the value of saliva as a monitoring tool in cancer treatment.

Despite numerous research studies on salivary biomarkers, saliva monitoring still is in its infancy. Streckfus et al. [87] observed pre-, peri- and post-chemotherapy variations in the salivary protein profile of an individual with Mantel cell lymphoma, suggesting the use of saliva for controlling disease progression.

### 2.9. Ovarian Cancer

Ovarian cancer is the most lethal gynaecological malignancy, with five- and 10-year relative survival rates of 45% and 35%, respectively [88]. Largely asymptomatic, ovarian cancer is usually diagnosed at the advanced stages (70% of new cases) and is characterised by the presence of massive peritoneal metastasis and ascites formation due to dissemination, which is directly related to poor prognosis [89]. Many efforts have been made to identify proteomic, genomic, metabolomic and transcriptomic biomarkers specific for ovarian cancer at tissue level and in body fluids [46,89,90,91], but the survival rate has not decreased in the last few decades [52], mainly because diagnosis at the early stage of the disease remains very low due to the fact that the current diagnostic biomarker (CA 125) is not very useful [92]. Attempting to improve early diagnosis, Lee et al. [14] analysed the salivary transcriptome, identifying four upregulated and 16 downregulated genes in the saliva of patients as compared to healthy controls. In the validation phase, seven downregulated mRNA biomarkers were significantly different between the saliva samples from the patients and controls. The logistic regression model of five validated biomarkers (*AGPAT1*, *B2M*, *BASP1*, *IER3*, *IL1B*) showed 85.7% sensitivity and 91.4% specificity.

Ławicki et al. evaluated CA 125 together with M-CSF levels with human epididymis protein 4 (HE4) in the plasma of patients with ovarian cancer [93]. There were statistically higher levels of M-CSF, CA 125 and HE4 in the cancer group with respect to the control group and healthy donors. Regarding the possible correlation between blood and saliva, Chen et al. [47] analysed CA 125 in serum and saliva samples from healthy women, patients with benign pelvic masses and patients with malignant pelvic tumours. Interestingly, no serum or saliva assay was positive in the healthy women. Nevertheless, significant differences were observed in the serum and salivary CA 125 values between the malignant and benign groups. Moreover, the saliva and serum CA 125 assays calculated in 16 patients with epithelial ovarian cancer had 81.3% and 93.8% sensitivity, respectively. The diagnostic efficiency and positive predictive value for salivary CA 125 were significantly higher than that in the serum assays. Indeed, there was a linear correlation between serum and salivary CA 125 levels. Overall, these results suggest the promising utility of saliva samples for managing ovarian cancer.

## 3. Future Perspectives

**Figure 2:**
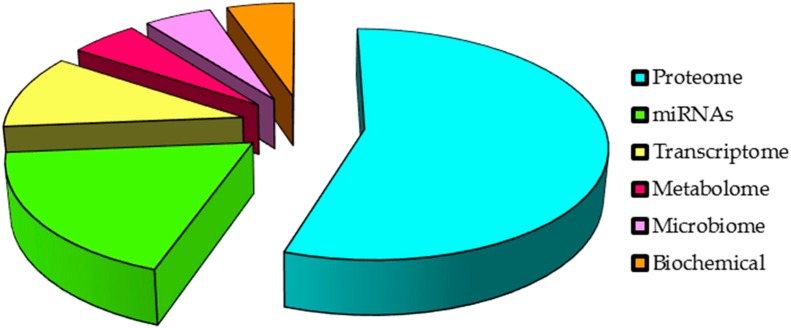
Leading “omics” in salivary biomarker development.

With all of the above, saliva represents a fluid enriched with tumour markers in patients with cancer remote/distant to the oral cavity (Figure 2). However, further research is needed in the different “omics” fields to consider saliva a liquid biopsy for a routine clinical usage. Regarding challenges in the field, a major limitation in saliva research is identifying specific disease biomarkers with high sensitivity and specificity. This literature review showed that many biomolecules have been identified as salivary biomarkers with significant differences among cancer and control groups; however, these biomarkers were not always validated. Multicentre studies with larger sample sizes and that include discovery, verification and validation phases are needed to validate the use of saliva. Robust studies comparing salivary biomarkers with other body fluids such as plasma or serum will also be important for determining the additional value of using saliva instead blood. There is also the urgent need for specific protocols and kits for saliva isolation and its posterior molecular analysis to avoid variations between the different approaches for quantifying salivary markers such as miRNAs. This includes a gold standard salivary normaliser for performing accurate quantification of certain biomarkers such as miRNAs.

**Figure 3:**
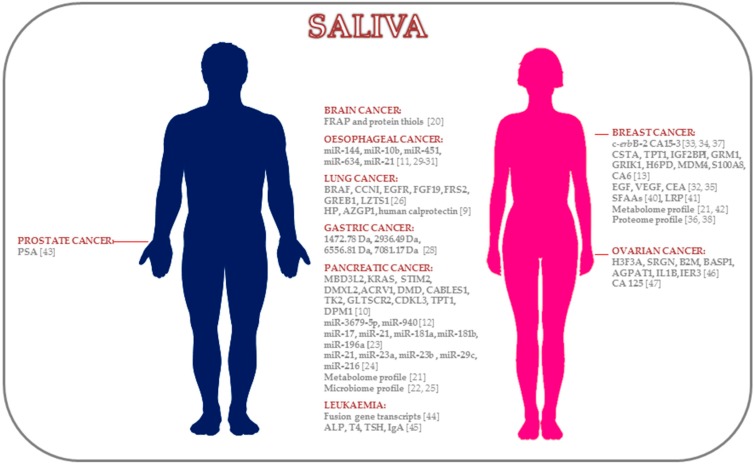
Summary of salivary diagnostic biomarkers for cancer distant to the oral cavity.

In addition, although proteomics leads the current investments in salivary biomarker development (Figure 3), high-throughput techniques such as next-generation sequencing (NGS) are currently the main techniques used in the field, increasing knowledge of miRNA and mRNA salivary biomarkers among the other RNAs. Despite the many efforts aimed made at discovering biomarkers for specific diseases, the discovery of new salivary markers and the validation of previously discovered saliva markers is required until salivary analysis has an actual clinical impact on cancer diagnosis and therapeutic monitoring. New perspectives must be directed towards finding specific biomarkers for each stage of disease, from the earliest to the latest, analysing the corresponding control population and including the treated and non-treated cancer groups. Regarding the value of saliva for monitoring systemic disease and taking into account the easy access to samples, there is an urgent need to develop robust studies to state its value in order to personalise the clinical management of patients with cancer. Overall, our review proves and reinforces the premise of saliva as a promising new liquid biopsy, confirming the valuable opportunity for current and future medical research.

## Figures and Tables

**Table 1:** Studies of saliva-based biomarkers.

Cancer Location	Study Design	Patients (No.)	Saliva Collection	Methods	Biomarkers	References
**Brain Cancer**	Biochemical	28 C, 32 B, 42 HC	Unstimulated	Spectrophotometric	FRAP, protein thiols	[20]
**Pancreatic Cancer**	Transcriptomic	DP: 12 C, 12 HC	Unstimulated	DP: Affymetrix U133 Plus 2.0 Array	MBD3L2, KRAS, STIM2, DMXL2, ACRV1, DMD, CABLES1, TK2, GLTSCR2, CDKL3, TPT1, DPM1	[10]
		VP: 30 C, 30 P, 30 HC		VP: RT-qPCR		
	Metabolomic	18 C, 87 HC	Unstimulated	CE-TOF-MS	Leucine with isoleucine tryptophan, valine, glutamic acid, phenylalanine, glutamine, aspartic acid	[21]
	Microbial	DP: 10 C, 10 HC	Unstimulated	DP: HOMIM	*Neisseria elongata*, *Streptococcus mitis*, *Granulicatella adiacens*	[22]
		VP: 28 C, 27 P, 28 HC		VP: RT-qPCR		
	miRNAs	30 C, 32 HC	NA	miScript miRNA PCR array human, RT-qPCR	miR-17, miR-21, miR-181a, miR-181b, miR-196a	[23]
	miRNAs	DP: 8 C, 8 HC	Stimulated (citric acid)	DP: Human miRNA Microarray, release 19.0 (Agilent)	miR-3679-5p, miR-940	[12]
		VP: 40 C, 20 B, 40 HC		VP: RT-qPCR		
	miRNAs	7 C, 4 P, 2 IPMN, 4 HC	NA	RT-qPCR using Fluidigm (Biomark)	miR-21, miR-23a, miR-23b, miR-29c, miR-216	[24]
	Microbial	8 C, 78 OD, 22 HC	NA	RT-qPCR	*Leptotrichia* sp. to *Porphyromonas* sp.	[25]
**Lung Cancer**	Proteomic	DP: 10 C, 10 HC	Unstimulated	DP: 2D-DIGE-MS	HP, AZGP1, human calprotectin	[9]
		VP: 26 C, 26 HC		VP: Western blotting, ELISA kits		
	Transcriptomic	DP: 10 C, 10 HC	Unstimulated	DP: Affymetrix HG U133 Plus 2.0 Array	BRAF, CCNI, EGFR, FGF19, FRS2, GREB1, LZTS1	[26]
		VP: 23 C, 64 HC		VP: RT-qPCR		
	Proteomic	21 C, 20 HC	Unstimulated	Surface-enhanced Raman spectroscopy	Amino acids, nucleic acid bases	[27]
**Gastric Cancer**	Proteomic	23 C, 18 HC	NA	MALDI-TOF-MS	1472.78 Da, 2936.49 Da, 6556.81 Da, 7081.17 Da	[28]
**Oesophageal Cancer**	miRNAs	DP: 8 EC, 4 HC	Stimulated (2% citric acid)	DP: RT-qPCR	miR-21	[29]
		VP: 32 C, 16 HC		VP: RT-qPCR		
	miRNAs	DP: 7 C, 3 HC	Stimulated (2% citric acid)	DP: Agilent microarray	miR-144, miR-10b, miR-451, miR-21	[11]
		VP: 39 C, 19 HC		VP: RT-qPCR		
	miRNAs	67 C, 50 HC	Stimulated (2% citric acid)	RT-qPCR	miR-144	[30]
	miRNAs	100 C, 50 HC	Stimulated (citric acid)	RT-qPCR	miR-21	[31]
**Breast Cancer**	Proteomic	52 active BC, 22 non-active BC, 33 HC	Stimulated (lemon juice when necessary)	ER-EIA (Abbott)	EGF	[32]
	Proteomic	12 C, 8 B, 15 HC	Stimulated (cube of paraffin)	EIA kits, ELISA kits (Oncogene Research)	CA15-3, c-*erb*B-2	[33]
	Proteomic	30 C, 44 B, 57 HC	Stimulated (gum base)	ELISA kits and EIA kits	c-*erb*B-2	[34]
	Proteomic	49 C, 49 HC	Unstimulated	ELISA kits	VEGF, EGF, CEA	[35]
	Proteomic	10 C, 10 B, 10 HC	Stimulated (paraffin or gum base)	IL-LC-MS/MS	40 protein profiles	[36]
	Proteomic	26 C, 35 HC	Unstimulated	EIA kits	CA15-3	[37]
	Proteomic	20 C, 10 HC	Stimulated (paraffin or gum base)	IL-LC-MS/MS	20 proteins (Stage IIa) 28 proteins (Stage IIb)	[38]
	Transcriptomic	DP: 10 C, 10 HC	Unstimulated	DP: Affymetrix HG U133 Plus 2.0 Array	CSTA, TPT1, IGF2BP1, GRM1, GRIK1, H6PD, MDM4, S100A8	[13]
		VP: 30 C, 63 HC		VP: RT-qPCR		
	Proteomic	DP: 10 C, 10 HC		DP: 2D-DIGE	CA6	
		VP: 30 C, 63 HC		VP: Western blotting		
	Metabolomic	30 C, 87 HC	Unstimulated	CE-MS	28 metabolites	[21]
	Proteomic	20 C, 10 HC	NA	LC-MS/MS	Protein profile	[39]
	Proteomic	27 C, 28 HC	Unstimulated	UPLC-MS	15 SFAAs	[40]
	Proteomic	16 C, 16 HC	Stimulated (gum base)	Gel electrophoresis and western blotting	Lung resistance protein	[41]
	Metabolomic	111 C, 61 HC	NA	UPLC-MS/MS	Polyamines	[42]
**Prostate Cancer**	Proteomic	11 high serum PSA prostate C, 20 low serum PSA prostate C	Stimulated (citrate-containing cotton)	ELISA	PSA	[43]
**Leukaemia**	Transcriptomic	7 C, 20 HC	Stimulated (citric acid)	RT-qPCR	BCR-ABL, PML-RARα, AML-ETO	[44]
	Biochemical	32 C, 115 HC	Unstimulated	Biochemical analysis, EIA, chemoluminescence	ALP, T4, TSH, IgA	[45]
**Ovarian Cancer**	Transcriptomic	DP: 11 C, 11 HC	Unstimulated	DP: Affymetrix HG U133 Plus 2.0 Array	H3F3A, SRGN, B2M, BASP1, AGPAT1, IL1B, IER3	[46]
		VP: 21 C, 35 HC		VP: RT-qPCR		
	Proteomic	92 B, 41 C, 55 HC	NA	Radioimmunoassay	CA 125	[47]

Abbreviations: DP, discovery phase; VP, validation phase; C, cancer; P, pancreatitis; HC, healthy controls; IPMN, intraductal papillary mucinous neoplasia; OD, other diseases; SFAAs, salivary free amino acids; PSA, prostate-specific antigen; VEGF, vascular endothelial growth factor; EGF, epidermal growth factor; CEA, carcinoembryonic antigen; NA, not available; FRAP, ferric acid reducing ability; RT-qPCR, reverse transcription–PCR; CE-TOF-MS, capillary electrophoresis and time-of-flight/mass spectrometry; HOMIM, Human Oral Microbe Identification Microarray; 2D-DIGE-MS, 2-dimensional difference gel electrophoresis and mass spectrometry; ELISA, enzyme-linked immunosorbent assay; MALDI-TOF-MS, matrix-assisted laser desorption ionisation time-of-flight/mass spectrometry; ER-EIA, oestrogen receptor enzyme immunoassay; IL-LC-MS/MS, isotopic labelling coupled with liquid chromatography tandem mass spectrometry; UPLC-MS/MS, ultra-performance liquid chromatography tandem mass spectrometry.
